# Is the RAND-36 an Adequate Patient-reported Outcome Measure to Assess Health-related Quality of Life in Patients Undergoing Bariatric Surgery?

**DOI:** 10.1007/s11695-021-05736-9

**Published:** 2021-11-02

**Authors:** Claire E. E. de Vries, Dennis J. S. Makarawung, Valerie M. Monpellier, Ignace M. C. Janssen, Steve M. M. de Castro, Ruben N. van Veen

**Affiliations:** 1 Department of Surgery, Obesity Center Amsterdam, OLVG West, Jan Tooropstraat 164, 1061 AE Amsterdam, the Netherlands; 2 Nederlandse Obesitas Kliniek (Dutch Obesity Clinic), Amersfoortseweg 43, 3712 Huis Ter Heide, the Netherlands

**Keywords:** Bariatric surgery, Patient-reported outcome measures, Patient-centered care, Health-related quality of life

## Abstract

**Purpose:**

The RAND-36 is the most frequently used patient-reported outcome measure (PROM) to evaluate health-related quality of life (HRQoL) in bariatric surgery. However, the RAND-36 has never been adequately validated in bariatric surgery. The purpose of this study was to validate the RAND-36 in Dutch patients undergoing bariatric surgery.

**Material and Methods:**

To validate the RAND-36, the following measurement properties were assessed in bariatric surgery patients: validity (the degree to which the RAND-36 measures what it purports to measure (HRQoL)), reliability (the extent to which the scores of the RAND-36 are the same for repeated measurement for patients who have not changed in HRQoL), responsiveness (the ability of the RAND-36 to detect changes in HRQoL over time).

**Results:**

Two thousand one hundred thirty-seven patients were included. Validity was not adequate due to the irrelevance of some items and response options, the lack of items relevant to patients undergoing bariatric surgery, and the RAND-36 did not actually measure what it was intended to measure in this study (HRQoL in bariatric surgery patients). Reliability was insufficient for the majority of the scales (the scores of patients who had not changed in HRQoL were different when the RAND was completed a second time (intraclass correlation coefficient (ICC) values 0.10–0.69)). Responsiveness was insufficient.

**Conclusion:**

The RAND-36 was not supported by sufficient validation evidence in patients undergoing bariatric surgery, which means that the RAND-36 does not adequately measure HRQoL in this patient population. Future research studies should use PROMs that are specifically designed for assessing HRQoL in patients undergoing bariatric surgery.

**Graphical abstract:**

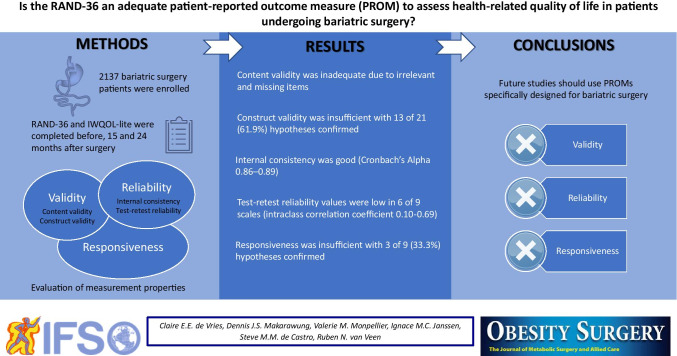

**Supplementary Information:**

The online version contains supplementary material available at 10.1007/s11695-021-05736-9.

## Introduction

The most effective treatment modality for severe obesity is bariatric surgery, which can lead to substantial improvements in patients’ health and well-being [1–3]. Although percent total weight loss (%TWL), morbidity, and mortality have often been the primary outcomes, they may not capture the impact of bariatric surgery on patients’ symptoms, functional and psychological aspects of health, and overall health-related quality of life (HRQoL) [4]. Analysis of HRQoL data can provide valuable information on the patient’s perspectives of bariatric surgery and can best be measured with patient-reported outcome measures (PROMs) [5]. High-quality PROMs provide a useful tool for clinical and research purposes. The quality of a PROM is determined by assessing measurement properties, including validity, reliability, and responsiveness [6]. If the measurement properties of a PROM are insufficient, the PROM will not reliably measure what it is supposed to measure, leading to uncertainties about the results.

While HRQoL is considered to be a key outcome in bariatric surgery, no consensus exists as to which PROMs should be used to assess HRQoL in bariatric surgery [7, 8]. A previous systematic review showed that 68 different PROMs were used in bariatric surgery studies [4, 9]. The RAND-36 was found to be one of the most frequently used measures in the bariatric surgery population [7, 8, 10–12]. The RAND-36 assesses generic HRQoL and is widely used in various health conditions [13]. It covers core health domains such as physical and mental health that is determined by both weight and other factors.

Although the RAND-36 is considered a reliable, valid, and responsive PROM to assess HRQoL in many other populations than patients undergoing bariatric surgery [14], it has only been validated for use in patients with obesity who were scheduled for bariatric surgery in a single institution in Bahrain [15]. Furthermore, two other studies showed some validation evidence in a population with severe obesity who received conservative treatment [16, 17]. The measurement properties of the RAND-36 for patients who undergo bariatric surgery are largely unknown, which is a major limitation to its use in research and clinical practice. In order to interpret the treatment effect of bariatric surgery using this PROM, it is essential that the RAND-36 is valid, reliable, and responsive to change in this specific population. The purpose of this study was to validate the RAND-36 in patients undergoing bariatric surgery.

## Methods

### Design and Study Population

The current study was a combination of a retrospective analysis of prospectively collected data and a prospective study.

For the retrospective analysis, patients were selected from the database of the Nederlandse Obesitas Kliniek (Dutch Obesity Clinic, NOK), which is the largest outpatient clinic for bariatric surgery in the Netherlands. All patients at the NOK were screened according to the International Federation of Surgery for Obesity (IFSO) criteria [18] and follow an interdisciplinary treatment program in addition to surgery [19]. Patients were selected if they underwent bariatric surgery before 2014 and if the RAND-36 results were available before surgery or at least at one follow-up moment after surgery. The data was previously used to assess the relationship between weight loss and HRQoL in patients who underwent bariatric surgery [20].

For the prospective part of the study, 125 patients who either started their treatment at the Nederlandse Obesitas Kliniek (NOK, Dutch Obesity Clinic) or who were one year post-operative were invited to participate in a test–retest study. Patients who were 18 years or older and who could read Dutch were included. In addition, patients and healthcare providers were sent a questionnaire about the RAND-36 to evaluate content validity, with up to two email reminders.

Ethical approval was obtained by the regional and local institutional review boards (registration number W17.138). Patients signed an online informed consent form prior to participation in the study. All collected patient data was coded to ensure subject privacy. The study was conducted in accordance with the Handbook for Good Clinical Research Practice of the World Health Organization and the Declaration of Helsinki principles.

### Data Collection

The following patient demographics were collected from the prospective database of the NOK: gender, age, weight, length, body mass index (BMI), and comorbidities (hypertension, diabetes mellitus, obstructive sleep apnea syndrome, hypercholesterolemia, and osteoarthritis) at baseline. HRQoL was routinely assessed in the treatment program. Since 2012, the RAND-36 has been used and the impact of weight on quality of life (IWQOL) lite was subsequently added. This treatment program was enrolled over the different clinics during 2012 and 2013. The questionnaires were administered at the preoperative screening and each year postoperatively. Furthermore, the 15 months follow-up of the questionnaires was chosen because the lifestyle group trajectory was up until 15 months, and HRQoL was evaluated at the end of this treatment program.

For the prospective study (test–retest), patients completed the RAND-36 twice: first as part of their regular treatment program and second at least 2 weeks after this first assessment. For the second questionnaire, an email with a URL that linked directly into a secure web-based application (Castor EDC) was sent to the participants of the test–retest study [21]. Up to two weekly reminders were sent. Data collection of the prospective study took place between April 2018 and May 2019.

### Measures

#### The RAND-36

The RAND-36 is a PROM that assesses general health in patients with different kinds of medical conditions and is one of the most widely used PROMs for assessing general health [22]. It contains 36 questions and eight scales: physical functioning, role limitations due to physical problems, bodily pain, general health, vitality, social functioning, role limitations due to emotional problems, and mental health. Two subscales can be generated from these eight scales: physical health summary (PHS) and mental health summary (MHS). Each scale has a total score that ranges from 0 (extremely poor) to 100 points (no complaint) [23]. The RAND-36 is different from the SF-36 in scoring algorithm (different scoring algorithms for two of the eight subscales).

#### The Impact of Weight on Quality of Life Questionnaire Lite

The IWQOL-lite is a disease (obesity) specific, 31-item PROM that assesses the impact of weight on quality of life in five domains: physical functioning, self-esteem, sexual life, public distress, and work [24]. This PROM showed sufficient validity and reliability in patients with obesity (Internal consistency, Cronbach’s alpha > 0.80; test–retest reliability, ICC > 0.81; discriminative validity, correlations with treatment-seeking status in patients with obesity) [25].

#### Analysis

Patient characteristics with regard to age, gender, BMI, comorbidities, and follow-up were described as the mean ± SD or by percentages. All analyses were performed with SPSS 25.0 for Windows (SPSS Inc. Chicago Illinois, USA) [26]. A two-tailed significance level of ≤ 0.05 was considered significant.

The COnsensus-based Standards for the selection of health status Measurement INstruments (COSMIN) standards for design requirements and preferred statistical methods was used for evaluating the measurement properties of the PROMs [27]. The following measurement properties were evaluated in bariatric surgery patients:

1. Validity, which refers to the degree to which the RAND-36 measures what it purports to measure (HRQoL) [28]. More specifically, the measurement properties content validity and construct validity were evaluated. In this study, content validity refers to whether bariatric surgery patients and healthcare providers consider the items of the RAND-36 relevant, comprehensive, and comprehensible to measure HRQoL in patients undergoing bariatric surgery [28]. Construct validity refers to whether the RAND-36 actually measures what it is intended to measure, i.e., HRQoL in patients undergoing bariatric surgery [28].
2. Reliability, which refers to the extent to which the scores of the RAND-36 are the same for repeated measurement for patients who have not changed [28]. In this regard, internal consistency and test–retest reliability were evaluated. In this study, internal consistency describes how reliably the items in the RAND-36 that are designed to measure the same aspect of HRQoL (e.g., physical functioning) actually do this [28]. Test–retest reliability measures whether the scores of the RAND-36 are the same when a patient whose HRQoL has not changed completes the RAND-36 the second time [28].
3. Responsiveness, which describes whether the RAND-36 is able to measure changes in HRQoL before and after bariatric surgery [28].

The definitions, interpretations, statistical tests, and quality criteria of the measurement properties are shown in the Supplementary Information, Table 1.

Content validity is considered the most important measurement property. Content validity was assessed by an online survey sent to patients and healthcare providers (bariatric physicians, bariatric surgeons, bariatric nurses, endocrinologists, psychologists, movement therapists, dieticians, and researchers). Patients were asked to give feedback on the comprehensiveness, comprehensibility, and relevance, while healthcare providers were asked to provide feedback on the comprehensiveness and relevance of the RAND-36.

## Results

A total of 2,137 patients completed the RAND-36 preoperatively or at least once postoperatively. The majority of patients were female (*n* = 1762, 82,5%), mean age was 46 SD 11 years, mean BMI preoperatively was 44.5 SD 5.8 kg/m^2^. Patient characteristics are displayed in Table 1. The RAND-36 was completed by 2074 patients (97.1%) 15 months postoperatively and by 1036 patients (48.5%) 24 months postoperatively.

**Table 1:** Demographics of included population at baseline (*n* = 2137), 15 months (*n* = 2093) and 24 months (*n* = 1079), adapted from Monpellier et al. 2017

Baseline	
Age, years, mean (SD)	46 (11)
Female, *n* (%)	1762 (82.5)
BMI baseline, kg/m^2^, mean (SD)	44.5 (5.8)
Diabetes Mellitus, *n* (%)	503 (23.5)
Hypertension, *n* (%)	838 (39.2)
Obstructive sleep apnea, *n* (%)	237 (11.1)
Hypercholesterolemia, *n* (%)	429 (20.1)
Osteoarthritis, *n* (%)	274 (12.8)
No comorbities, *n* (%)	925 (43.3)
Follow-up	
15 M BMI, kg/m^2^, mean (SD)	30.7 (5.1)
24 M BMI, kg/m^2^, mean (SD)	30.7 (5.2)
15 M ΔBMI, kg/m^2^, mean (SD)	13.8 (4.1)
24 M ΔBMI, kg/m^2^, mean (SD)	13.9 (4.3)
15 M %TWL, mean (SD)	31.0 (7.9)
24 M %TWL, mean (SD)	31.1 (8.4)

*15 M*, 15 months follow-up; *24 M*, 24 months follow-up; *BMI*, body mass index; *ΔBMI*, change in BMI; *%TWL*, % total weight loss

### Validity

#### Content Validity

The online survey was completed by 53 patients and 50 healthcare providers. The results of the online survey are shown in Table 2. The majority of the patients (92.5%) and healthcare providers (76.0%) noted that most items and response options were relevant to measure HRQoL, but not as relevant for patients undergoing bariatric surgery (73.6% of the patients and 68% of the healthcare providers). The recall periods of the questions were not appropriate according to 47.0% of the patients and 52.0% of the caregivers. For example, one question has a recall period of 1 year, which does not always reflect the timeframe that changes have occurred during the total weight loss journey. The majority of the healthcare providers (52.0%) and a selection of the patients (20.8%) indicated that key concepts of patients undergoing bariatric surgery were missing in the RAND-36. Patients reported that items on issues such as eating behavior, body image, obesity-specific symptoms, and symptoms after surgery were missing. Furthermore, healthcare providers stated that the RAND-36 lacks items on aspects important to patients undergoing bariatric surgery including excess skin, stigma, sexual functioning, work life, and appearance. Patients generally did not have any problems with the comprehensibility of the items. However, some patients asked for shorter sentences and simplified language. Thus, content validity of the RAND-36 was not sufficient for patients undergoing bariatric surgery.

**Table 2:** Content validity of the RAND-36 (online survey)

		Patients (*N* = 53, *N* (%))	Caregivers (*N* = 50, *N* (%))
Relevance	*The included items are relevant for the construct of interest*	49 (92.5)	38 (76.0)
	*The included items are relevant for the target population of interest*	39 (73.6)	34 (68.0)
	*The included items are relevant for the context of use of interest*	46 (86.8)	34 (68.0)
	*The response options are appropriate*	41 (77.4)	40 (80.0)
	*The recall period is appropriate*	25 (47.2)	26 (52.0)
Comprehensiveness	*There are no key concepts missing*	42 (79.2)	24 (48.0)
Comprehensibility	*The PROM instructions are understood by the population of interest as intended*	50 (94.3)	
	*The PROM items and response options are understood by the population of interest as Intended*	46 (86.8)	
	*The PROM items are appropriately worded*	48 (90.6)	
	*The response options match the question*	47 (88.7)	

#### Construct Validity

Only 13 of the 21 hypotheses (61.9%) were confirmed (Supplementary Information, Table 2). Therefore, construct validity was not considered sufficient.

#### Convergent and Divergent Validity

For convergent and divergent, the majority of the RAND-36 subscales and IWQOL lite subscales measuring the same construct had moderate to high correlations, and scales measuring a different construct had lower correlations. However, for discriminative validity, none of the a priori hypotheses were confirmed by the data. The RAND-36 scales could not adequately discriminate between gender, comorbidities, age or BMI.

### Reliability

#### Internal Consistency

Internal consistency was good with Cronbach’s alpha values ranging from 0.86 to 0.89 for the different subscales of the RAND-36.

#### Test–Retest Reliability

The results of test–retest reliability are shown in Table 3. Test–retest reliability was not sufficient in six of the nine scales, only the physical functioning, general health perceptions, and health change scales had sufficient ICC values higher than 0.70.

**Table 3:** Test–retest reliability of the RAND-36

RAND-36 subscale	ICC value	95% confidence interval
Physical functioning	0.880	0.827–0.918
Role limitations due to physical problems	0.624	0.489–0.730
Bodily pain	0.687	0.568–0.778
General health	0.857	0.794–0.902
Vitality	0.096	0.000–0.258
Social functioning	0.630	0.495–0.734
Role limitations due to emotional problems	0.430	0.240–0.589
Mental health	0.422	0.003–0.671
Health change	0.868	0.810–0.909

*ICC*, intraclass correlation coefficient

### Responsiveness

For responsiveness, three of the nine hypotheses (33.3%) were confirmed by the data (Supplementary Information, Table 3). The changes on the RAND-36 subscales were only weakly or moderately correlated (< 0.50) with changes on the IWQOL lite subscales measuring the same construct (exception physical functioning (*r* > 0.50, *p* < 0.001). The RAND-36 subscales correlated weakly (*r* < 0.30) with %TWL and change in BMI after surgery. The change scores of the RAND-36 could not discriminate between subgroups (gender, age, BMI, and comorbidities).

## Discussion

While the assessment of the validity of measures such as blood pressure is common, the awareness of the importance of validation evidence of PROMs is less common. This study assessed the measurement properties of the RAND-36 in a large population of patients who underwent bariatric surgery. The quality of a PROM is crucial when used in research or clinical practice and should be evaluated by assessing measurement properties [6]. It is important to consider that in case of insufficient measurement properties the PROM is not adequate for its purpose.

This study only demonstrated evidence of sufficient internal consistency, meaning good interrelatedness among the items of the RAND-36. The most important result was that content validity was not adequate due to the irrelevance of some items and response options, and the lack of other items that are relevant to patients undergoing bariatric surgery. Resultant low test–retest reliability values, insufficient construct validity, and responsiveness limit the ability of the RAND-36 to be used in bariatric surgery. These results indicate that the RAND-36 lacks items important to patients undergoing bariatric surgery and is limited in its ability to measure HRQoL and detect relevant changes in HRQoL after bariatric surgery. Furthermore, the scores of the RAND-36 in patients undergoing bariatric surgery may not be reliable.

Content validity is considered the most important measurement property and refers to the extent to which the items of the RAND-36 measure all relevant aspects of HRQoL in the bariatric population. Nearly one-third of the participants noted that a number of items and response options were irrelevant for patients undergoing bariatric surgery. Approximately half of the patients and healthcare providers answered that the recall period was not adequate for this population. Irrelevant content can lead to insufficiency to measure relevant changes over time and inconsistency among patients in answering the questions. This may be reflected in the insufficient results with regards to test–retest reliability and responsiveness in this study.

Another issue with the content validity was that participants noted that key concepts of HRQoL in bariatric surgery patients were missing in the RAND-36. The RAND-36 was developed in a general population, and, therefore, the items lack particular issues relevant to bariatric surgery patients, such as eating behavior, stigma, sexual functioning, appearance, body image, and excess skin. Some of these issues add substantially to the well-being of patients with obesity or undergoing bariatric surgery.

Interestingly, there were weak correlations between BMI or %TWL and RAND-36 scores in this study, which means that patients with higher BMI or less %TWL were not necessarily the patients with lower HRQoL scores. Only the physical functioning scales of the RAND-36 correlated strongly with the IWQOL-lite and could discriminate between patients with different BMI or %TWL. To adequately assess the effect of bariatric surgery, an effect of BMI or %TWL should be reflected in change in HRQoL. Other questionnaires specifically developed for people living with obesity, such as the IWQOL-Lite and BODY-Q, demonstrated strong evidence for discriminative validity in patients with different BMI categories and differences in weight loss [29–34]. While previous clinical studies (not clinimetric/psychometric studies) showed associations or correlations between BMI or %TWL and the RAND-36 [35, 36], we tested a priori hypotheses that specified the expected relative magnitude of the differences between different BMI groups and correlations with %TWL in this study. The interpretation of these results is different in that we did not test statistical significance, but whether the RAND-36 truly measured changes in HRQoL and whether it measured the right amount of change [27].

The results of this study contradict the only evidence of validity of the RAND-36 in patients who were scheduled for bariatric surgery [14]. In our study, we did not repeat the same analyses, but assessed the additional measurement properties in patients undergoing bariatric surgery. The major limitation of the study by Al Amar is that they did not assess the most important measurement property, content validity [14]. The use of a PROM in a different patient population than the population for which it was developed requires new supporting evidence of content validity. Moreover, the additional measurement properties (construct validity, reliability, and responsiveness) are important to ensure that studies adequately evaluate treatment effects as in bariatric surgery.

Strengths of the study include the large number of participants, the inclusion of patients in the evaluation of content validity, and the generation of a priori hypotheses to assess construct validity. Previous studies included only smaller samples of patients with obesity. However, there were some limitations to this study. First, part of the study data was retrospective and only included data of patients that filled out the questionnaires (even though all patients in the treatment program were expected to complete the questionnaires). This may have introduced selection bias to this study. Furthermore, the follow-up rate at 24 months after surgery was less than 50%, which may have introduced further bias to the results of responsiveness. Second, the content validity of the RAND-36 was assessed with an online survey with patients and healthcare providers. Qualitative methods to assess content validity would have improved the quality of evidence of this measurement property. Third, this study was performed in the Netherlands using the Dutch RAND-36. Different language versions of the RAND-36 may show different results.

Bariatric surgery can be evaluated by many different outcomes, including clinical endpoints such as weight loss and improvements in comorbidities, and patient-reported outcomes (PROs) such as HRQoL. Even though the SF-36 and RAND-36 are frequently chosen measures in bariatric surgery, these PROMs are designed for general use. They allow for comparison across different patient groups, but they lack sensitivity to measure changes in patients undergoing bariatric surgery. This means that the use of the RAND-36 alone may not be sufficient to assess the effects of bariatric surgery from the patients’ perspective. The RAND-36 is useful to compare patients undergoing bariatric surgery with other patient populations to demonstrate the burden of disease, but a PROM specifically designed for assessing HRQoL in bariatric surgery patients should be used to discriminate at another level among subgroups of these patients.

## Conclusion

The RAND-36 was not supported by sufficient validation evidence in patients undergoing bariatric surgery, which means that the RAND-36 does not adequately measure HRQoL in this patient population. Future research studies should use PROMs that are specifically designed for assessing HRQoL in patients undergoing bariatric surgery.

## Supplementary Information

Below is the link to the electronic supplementary material.

Supplementary file1 (DOCX 25 KB)

## References

[1] Buchwald H, Avidor Y, Braunwald E (2004). Bariatric surgery: a systematic review and meta-analysis. JAMA.

[2] Colquitt JL, Pickett K, Loveman E, et al. Surgery for weight loss in adults (Cochrane Review). Cochrane Database Syst Rev. 2014;8:CD003641. 10.1002/14651858.CD003641.pub4 PMC9028049 25105982

[3] Christou NV, Sampalis JS, Liberman M (2004). Surgery decreases long-term mortality, morbidity, and health care use in morbidly obese patients. Ann Surg.

[4] Coulman KD, Abdelrahman T, Owen-Smith A (2013). Patient-reported outcomes in bariatric surgery: a systematic review of standards of reporting. Obes Rev.

[5] Patrick DL, Burke LB, Powers JH, et al. Patient-reported outcomes to support medical product labeling claims: FDA perspective. Value Health. 2007;10 Suppl 2:S125–37. 10.1111/j.1524-4733.2007.00275.x 17995471

[6] Terwee CB, Bot SDM, de Boer MR (2007). Quality criteria were proposed for measurement properties of health status questionnaires. J Clin Epidemiol.

[7] Brethauer SA, Kim J, el Chaar M (2015). Standardized outcomes reporting in metabolic and bariatric surgery. Surg Obes Relat Dis.

[8] Coulman KD, Hopkins J, Brookes ST (2016). A core outcome set for the benefits and adverse events of bariatric and metabolic surgery: the BARIACT project. PLoS Med.

[9] de Vries CEE, Kalff MC, Prinsen CAC, et al. Recommendations on the most suitable quality-of-life measurement instruments for bariatric and body contouring surgery: a systematic review. Obes Rev. 2018;10:1395–411. 10.1111/obr.12710 29883059

[10] Hachem A, Brennan L (2016). Quality of life outcomes of bariatric surgery: a systematic review. Obes Surg.

[11] Jumbe S, Bartlett C, Jumbe SL (2016). The effectiveness of bariatric surgery on long term psychosocial quality of life - a systematic review. Obes Res Clin Pr.

[12] Raaijmakers LC, Pouwels S, Thomassen SE (2017). Quality of life and bariatric surgery: a systematic review of short- and long-term results and comparison with community norms. Eur J Clin Nutr.

[13] McHorney CA, Ware JE, Raczek AE (1993). The MOS 36-Item Short-Form Health Survey (SF-36): II. Psychometric and clinical tests of validity in measuring physical and mental health constructs. Med Care.

[14] Hays RD, Morales LS (2001). The RAND-36 measure of health-related quality of life. Ann Med.

[15] Al Amer R, Al Khalifa K, Alajlan SA (2018). Analyzing the psychometric properties of the Short Form-36 quality of life questionnaire in patients with obesity. Obes Surg.

[16] Corica F, Corsonello A, Apolone G (2006). Construct validity of the Short Form-36 Health Survey and its relationship with BMI in obese outpatients. Obes (Silver Spring).

[17] Karlsen TI, Tveita EK, Natvig GK (2011). Validity of the SF-36 in patients with morbid obesity. Obes Facts.

[18] Fried M, Yumuk V, Oppert JM (2014). Interdisciplinary European guidelines on metabolic and bariatric surgery. Obes Surg.

[19] Tettero OM, Aronson T, Wolf RJ (2018). Increase in physical activity after bariatric surgery demonstrates improvement in weight loss and cardiorespiratory fitness. Obes Surg.

[20] Monpellier VM, Antoniou EE, Aarts EO (2017). Improvement of health-related quality of life after Roux-en-Y gastric bypass related to weight loss. Obes Surg.

[21] Castor EDC. 2019. Available from: https://castoredc.com.

[22] VanderZee KI, Sanderman R, Heyink JW (1996). Psychometric qualities of the RAND 36-Item Health Survey 1.0: a multidimensional measure of general health status. Int J Behav Med.

[23] Ware JE, Sherbourne CD (1992). The MOS 36-Item Short-Form Health Survey (Sf-36): I. conceptual framework and item selection. Med Care.

[24] Kolotkin RL, Crosby RD, Kosloski KD (2001). Development of a brief measure to assess quality of life in obesity. Obes Res.

[25] Forhan M, Vrkljan B, MacDermid J, et al. A systematic review of the quality of psychometric evidence supporting the use of an obesity-specific quality of life measure for use with persons who have class III obesity: Diagnostic in Obesity and Complications. Obes Rev. 2010;3:222–8. 10.1111/j.1467-789X.2009.00612.x 19493301

[26] IBM Corp. Released 2017. IBM SPSS Statistics for Windows, Version 25.0. Armonk, NY: IBM Corp

[27] Mokkink LB, Terwee CB, Patrick DL (2010). The COSMIN checklist for assessing the methodological quality of studies on measurement properties of health status measurement instruments: an international Delphi study. Qual Life Res.

[28] Mokkink LB, Terwee CB, Patrick DL (2010). The COSMIN study reached international consensus on taxonomy, terminology, and definitions of measurement properties for health-related patient-reported outcomes. J Clin Epidemiol.

[29] Klassen AF, Kaur M, Breitkopf T (2018). Using the BODY-Q to understand impact of weight loss, excess skin, and the need for body contouring following bariatric surgery. Plast Reconstr Surg.

[30] Klassen AF, Cano SJ, Alderman A (2016). The BODY-Q: A patient-reported outcome instrument for weight loss and body contouring treatments. Plast Reconstr Surg - Glob Open.

[31] Kolotkin RL, Crosby RD, Pendleton R (2003). Health-related quality of life in patients seeking gastric bypass surgery vs non-treatment-seeking controls. Obes Surg.

[32] Kolotkin RL, Kim J, Davidson LE (2018). 12-year trajectory of health-related quality of life in gastric bypass patients versus comparison groups. Surg Obes Relat Dis.

[33] Kolotkin RL, Davidson LE, Crosby RD (2012). Six-year changes in health-related quality of life in gastric bypass patients versus obese comparison groups. Surg Obes Relat Dis.

[34] Kolotkin RL, Crosby RD, Gress RE (2009). Two-year changes in health-related quality of life in gastric bypass patients compared with severely obese controls. Surg Obes Relat Dis.

[35] Antonsson T, Wennersten A, Sörensen K (2021). Differences in health-related quality of life after gastric bypass surgery: a cross-sectional study. Obes Surg.

[36] Raoof M, Szabo E, Karlsson J (2020). Improvements of health-related quality of life 5 years after gastric bypass. What is important besides weight loss? A study from Scandinavian Obesity Surgery Register. Surg Obes Relat Dis.

