# Supplementary material for: Is the RAND-36 an Adequate Patient-reported Outcome Measure to Assess Health-related Quality of Life in Patients Undergoing Bariatric Surgery?
Source: Obes Surg. 2021 Nov 2;32(1):48–54. doi: 10.1007/s11695-021-05736-9 (PMC8752557; doi:10.1007/s11695-021-05736-9)
Supplement: Supplementary file 1 — Supplementary file1 (DOCX 25 KB) [file 11695_2021_5736_MOESM1_ESM.docx]

**Supplementary material**

**Table 1.** Definition of measurement properties and quality criteria

| **Measurement property** | **Definition^a^** | **Interpretation** | **Statistical test^b^** | **Quality criteria^c^** |
| --- | --- | --- | --- | --- |
| Content validity | The degree to which the content of a health-related PROM is an adequate reflection of the construct to be measured | The items in the RAND-36 should be considered relevant, comprehensive and comprehensible by healthcare providers and patients to measure HRQoL in patients undergoing bariatric surgery | Feedback from patients and healthcare providers on the comprehensiveness, comprehensibility and relevance | The target population and healthcare providers consider the items relevant, comprehensive and comprehensible |
| Internal consistency | The degree of interrelatedness among the items | The items in each RAND-36 scale that are meant to measure the same construct (e.g. physical functioning), should produce similar scores | Cronbach’s alpha coefficients | Cronbach’s alpha value of 0.70 and higher |
| Tet-retest reliability | The extent to which scores for patients who have not changed are the same for repeated measurement over time | The scores of the RAND-36 should be the same when a patient whose HRQoL has not changed completes the RAND-36 the second time | Intraclass correlation coefficients (ICC), using a two‐way random effects model | ICC values of ≥0.70 |
| Construct validity | The degree to which the scores of a health-related PROM are consistent with hypotheses based on the assumption that the health-related PROM validly measures the construct to be measured | The RAND-36 should actually measure what it is intended to measure, i.e. HRQoL in patients undergoing bariatric surgery | Predefined hypotheses (**Supplementary information, Table 2**) regarding:  1. The relationships of the RAND-36 to scores of the IWQOL-Lite that measure the same construct (i.e., divergent/convergent validity) (Spearman’s or Pearson correlation coefficients)  2. The differences in scoring between relevant groups (i.e., discriminative validity) (t-test or Mann-Whitney U test (depending on normality) | At least 75% of the results in concordance with the a priori hypotheses  Correlation coefficient values below 0.3 were considered low, between 0.3 and 0.6 moderate, and above 0.6 high |
| Responsiveness | The ability of a health-related PROM to detect change over time in the construct to be measured | The RAND-36 to should be able to measure changes in HRQoL before and after bariatric surgery | Predefined hypotheses (**Supplementary information, Table 3)** regarding changes in the RAND-36 were compared to changes in the IWQOL-lite (Pearson or Spearman’s correlation coefficients) | At least 75% of the results in concordance with the a priori hypotheses or correlation with changes on PROMs measuring the same construct was >0.50 |

^a^Definition of properties adapted from Mokkink et al. 2010 and ^b^ statistical tests adapted from Mokkink et al. 2010  ^c^quality criteria adapted from Terwee et al. 2007

*PROM*  patient-reported outcome measure, *HRQoL* health-related quality of life, *ICC* intraclass correlation coefficient, *IWQOL-Lite* impact of weight on quality of life -Lite

**Table 2.** Hypotheses of construct validity.

|  | **Hypothesis** | **Results** | **Hypothesis confirmed** |
| --- | --- | --- | --- |
|  | A high correlation (≥ 0.6) between RAND-36 physical functioning and IWQoL-Lite physical functioning | 0.724, p<0.001 | Yes |
|  | A high correlation (≥ 0.6) between RAND-36 social functioning and IWQoL-Lite public distress | 0.318*, p<0.001 | No |
|  | A high correlation (≥ 0.6) between RAND-36 physical health summary (PHS) scores and IWQoL-Lite physical | 0.683, p<0.001 | Yes |
|  | A moderate correlation (≥ 0.4) between RAND-36 role limitations due to physical problems and IWQoL-Lite physical | 0.494, p<0.001 | Yes |
|  | A moderate correlation (≥ 0.4) between RAND-36 role limitations due to physical problems and IWQoL-Lite work | 0.479*, p<0.001 | Yes |
|  | A moderate correlation (≥ 0.4) between RAND-36 social functioning and IWQoL-Lite self-esteem | 0.413*, p<0.001 | Yes |
|  | A moderate correlation (≥ 0.4) between RAND-36 RAND-36 mental health summary (MHS) scores and IWQoL-Lite self-esteem | 0.470, p<0.001 | Yes |
|  | A moderate correlation (≥ 0.4) between RAND-36 vitality and IWQoL-Lite physical functioning | 0.462, p<0.001 | Yes |
|  | A stronger correlation of the RAND-36 mental health summary (MHS) compared with IWQoL Lite public distress to the correlation with RAND-36 role limitations due to emotional problems and IWQoL Lite self-esteem. This difference will be at least 0.05 higher. | 0.338, p<0.001  0.283*, p<0.001 | Yes |
|  | A stronger correlation of the RAND-36 PHS with the IWQoL Lite physical compared to the correlation of the RAND-36 MHS and the other subscales of the IWQoL Lite. This difference should be at least 0.05 higher. | RAND physical, IWQOL physical (0.724, p<0.001)  RAND MHS, IWQOL physical (0.462, p<0.001)  RAND MHS, IWQOL Self-esteem (0.470, p<0.001)  RAND MHS, IWQOL Public Distress (0.338, p<0.001)  RAND MHS, IWQOL Work (0.530, p<0.001)  RAND MHS, IWQOL Sexual (0.392*, p<0.001) | Yes |
|  | A stronger correlation of the RAND-36 role limitations due to physical problems with IWQoL Lite work to the correlation with RAND-36 role limitations due to emotional problems and IWQoL Lite work. This difference will be at least 0.05 higher. | RAND physical, IWQOL work (0.479*, p<0.001)  RAND emotional, IWQOL Work (0.366*, p<0.001) | Yes |
|  | Low correlations ( ≤ 0.3) between RAND-36 bodily pain and IWQoL-Lite self-esteem. | RAND bodily pain, IWQOL self esteem (0.228, p<0.001) | Yes |
|  | Low correlations ( ≤ 0.3) between RAND-36 subscales and IWQoL-Lite sexual life. | RAND emotional, IWQOL sexual 0.218*, p<0.001  RAND social, IWQOL sexual 0.355*, p<0.001  RAND vitality, IWQOL sexual 0.319*, p<0.001  RAND physical, IWQOL sexual 0.281*, p<0.001  RAND mental health, IWQOL sexual 0.355*, p<0.001  RAND bodily pain, IWQOL sexual 0.241*, p<0.001  RAND general health, IWQOL sexual 0.226*, p<0.001  RAND health change, IWQOL sexual 0.155*, p<0.001  RAND physical role, IWQOL sexual 0.268*, p<0.001 | No |
|  | Low correlations ( ≤ 0.3) between RAND-36 subscales and IWQoL-Lite work. | RAND emotional, IWQOL work 0.366*, p<0.001  RAND social, IWQOL work 0.473*, p<0.001  RAND vitality, IWQOL work 0.411, p<0.001  RAND physical, IWQOL work 0.415, p<0.001  RAND mental health, IWQOL work 0.440, p<0.001  RAND bodily pain, IWQOL work 0.371, p<0.001  RAND general health, IWQOL work 0.353, p<0.001  RAND health change, IWQOL work 0.185*, p<0.001  RAND physical role, IWQOL work 0.479, p<0.001 | No |
|  | A higher number of comorbidities is associated with lower RAND-36 total score. | PHS -0.178*, p<0.001  MHS -0.070*, p=0.001 | Yes |
|  | A mean difference of >10 points on a scale from 0 to 100 on the RAND-36 between patients with comorbidities and without comorbidities. | RAND vitality -2.05, p=0.012  RAND physical -7.80, p<0.001  RAND mental health 0.14, p=0.856  RAND pain -7.11, p<0.001  RAND general health -4.71, p<0.001  RAND PHS -6.91, p<0.001  RAND MHS -1.95, p=0.022  RAND emotional p=0.017  RAND social p=0.246  RAND health change 0.052  RAND physical role p<0.001 | No |
|  | Low correlation ( ≤ 0.5) between RAND-36 scores and age. | RAND role emotional, age -0.038*, P=0.078  RAND social, age -0.027*, p=0.207  RAND vitality, age -0.034, P=0.114  RAND physical, age -0.242, p<0.001  RAND mental, age 0.034, p=0.112  RAND pain, age -0.161, P<0.001  RAND general, age 0.022, P=0.320  RAND health change, age -0.072*, P=0.001  RAND physical role, age -0.109*, p<0.001 | Yes |
|  | A mean difference of ≥10 points on a scale from 0 to 100 on the RAND-36 between men and woman. | RAND vitality 2.25, p=0.033  RAND physical 4.77, p=0.001  RAND mental 2.51, p=0.010  RAND pain 5.58, p<0.001  RAND general health -0.80, p=0.456  RAND PHS 3.14, p=0.009  RAND MHS 2.78, p=0.007  RAND emotional 3.12, p=0.279  RAND social 3.25, p=0.035  RAND health change 2.12, p=0.077  RAND physical role 3.02, p=0.169 | No |
|  | A mean difference of <10 points on a scale from 0 to 100 on the RAND-36 between age<40 and age>40. | RAND vitality 1.71, p=0.058  RAND physical 8.98, p<0.001  RAND mental -1.37, p=0.097  RAND pain 6.48, p<0.001  RAND general health -1.78, p=0.052  RAND PHS 4.58, p<0.001  RAND mental 0.50, p=0.598  RAND emotional role 1.94, p=0.621  RAND social -0.30, p=0.828  RAND health change 2.98, p=0.013  RAND physical role 4.63, p=0.021 | No |
|  | A mean difference of >10 points on a scale from 0 to 100 on the RAND-36 between BMI<50 and BMI≥50. | RAND vitality -1.24, p=0.24  RAND physical 9.73, p=<0.001  RAND mental 1.23, p=0.20  RAND pain 2.19, p=0.003  RAND general health 1.98, p=0.188  RAND PHS 4.43, p=0.056  RAND MHS 1.44, p=0.048  RAND emotional role 1.64, p=0.411  RAND social 4.12, p=0.020  RAND health change 1.63, p=0.150  RAND physical role 3.80, p=0.139 | No |
|  | A high correlation (≥ 0.6) between RAND-36 scales physical functioning, role limitations due to physical problems, bodily pain, general health and vitality and patients with BMI≥50. | RAND vitality, BMI 0.040, p=0.062  RAND physical, BMI -0.158, P<0.001  RAND pain, BMI -0.029, p=0.182  RAND general health, BMI -0.009, p=0.669  RAND physical role, BMI 1,000, p=0.356 | No |

**Table 3.** Hypotheses of responsiveness

|  | **Hypothesis** | **Results** | **Hypothesis confirmed** |
| --- | --- | --- | --- |
|  | A positive moderate correlation (≥ 0.5) between changes in the scores comparing the RAND-36 and the IWQoL-Lite | The changes on the RAND-36 subscales were only weakly or moderately correlated (<0.50) with changes on the IWQOL-Lite subscales measuring the same construct (exception physical functioning (r >0.50, p<0.001) | No |
|  | Change scores of the RAND-36 will be ≥ 0.1 lower compared with the change scores of the IWQoL-Lite | 15 M: RAND emotional role 0.10, RAND social 0.15, RAND vitality 0.17, RAND physical 0.24, RAND mental 0.07, RAND pain 0.19, RAND general health 0.28, RAND health change 0.57, RAND physical role 0.40, RAND PHS 0.28, RAND MHS 0.12  IWQOL physical 0.47, IWQOL self-esteem 0.39, IWQOL sexual 0.29, IWQOL public distress 0.32, IWQOL work 0.24  24 M: RAND emotional role 0.07, RAND social 0.12, RAND vitality 0.14, RAND physical 0.34, RAND mental 0.05, RAND pain 0.17, RAND general health 0.27, RAND health change 0.42, RAND physical role 0.28, RAND PHS 0.27, RAND MHS 0.09  IWQOL physical 0.47, IWQOL self-esteem 0.38, IWQOL sexual 0.28, IWQOL public distress 0.32, IWQOL work 0.24 | No |
|  | Change scores of the RAND-36 PHS will be ≥ 0.1 higher compared with the change scores of the RAND-36 MHS | 15 M: PHS 0.28, MHS 0.12  24 M: PHS 0.26, MHS 0.09 | Yes |
|  | The highest change score on physical functioning of the RAND-36 | 15 M: Physical functioning 0.35, Health change 0.57  24 M: Physical functioning 0.34, Health change 0.47 | No |
|  | The lowest change score on the RAND-36 MHS | 15 M: Mental health summary 0.12, Mental health 0.07  24 M: Mental health summary, 0.09, Mental health 0.05 | No |
|  | A mean difference of <10 points on a scale from 0 to 100 in change scores on the RAND-36 between men and woman | 15 M: RAND emotional role -2.53, p=0.32  RAND social -0.67, p=0.60  RAND vitality 0.65, p=0.85  RAND physical -2.80, p=0.02  RAND mental -0.81, p=0.41  RAND pain -2.89, p=0.13  RAND general health 0.23, p=0.95  RAND health change -1.78, p=0.15  RAND physical role -2.89, p=0.35  RAND PHS -1.93, p=0.12  RAND MHS -0.84, p=0.45  24 M: RAND emotional role 1.23, p=0.68  RAND social 1.46, p=0.67  RAND vitality 4.21, p=0.03  RAND physical -3.91, p=0.07  RAND mental 0.15, p=0.76  RAND pain -1.94, p=0.52  RAND general health 3.32, p=0.15  RAND health change 0.41, p=0.97  RAND physical role -2.19, p=0.59  RAND PHS -1.18, p=0.75  RAND MHS 1.76, p=0.60 | Yes |
|  | A mean difference of <10 points on a scale from 0 to 100 in change scores on the RAND-36 between age<40 and age>40 | 15 M: RAND emotional role 0.007, p=0.33  RAND social 1.75, p=0.36  RAND vitality -3.42, p=0.003  RAND physical -2.59, p=0.02  RAND mental 0.73, p=0.54  RAND pain -0.30, p=0.57  RAND general health 0.06, p=0.83  RAND health change -2.62, p=0.38  RAND physical role -0.29, p=0.83  RAND PHS -0.78, p=0.42  RAND MHS -0.23, p=0.84  24 M: RAND emotional role 0.47, p=0.76  RAND social -3.22, p=0.13  RAND vitality -4.84, p=0.004  RAND physical -3.05, p=0.16  RAND 1.01, p=0.40  RAND pain -4.13, p=0.01  RAND general health -0.86, p=0.92  RAND health change -6.87, p=0.02  RAND physical role -3.25, p=0.41  RAND PHS -2.82, p=0.11  RAND MHS -1.64, p=0.31 | Yes |
|  | A mean difference of >10 points on a scale from 0 to 100 in change scores on the RAND-36 between patients with comorbidities and without comorbidities | 15 M: RAND emotional role 2.95, p=0.20  RAND social -0.38, p=0.87  RAND vitality 1.71, p=0.08  RAND physical 2.03, p=0.05  RAND mental -1.25, p=0.09  RAND pain 1.86, p=0.09  RAND general health 2.95, p=0.004  RAND health change 3.47, p=0.006  RAND physical role 3.44, p=0.06  RAND PHS -2.57, p=0.009  RAND MHS 0.76, p=0.49  24 M: RAND emotional role 5.23, p=0.11  RAND social 0.94, p=0.97  RAND vitality 2.98, p=0.09  RAND physical 2.17, p=0.22  RAND mental 0.04, p=0.54  RAND pain 3.82, p=0.02  RAND general health 2.06, p=0.23  RAND health change 5.93, p=0.02  RAND physical role 6.19, p=0.04  RAND PHS 3.56, p=0.02  RAND MHS 2.30, p=0.17 | No |
|  | A mean difference of >10 points on a scale from 0 to 100 in change scores on the RAND-36 between patients between BMI<50 and patients with a BMI≥50 | 15 M: RAND emotional role -5.15, p=0.04  RAND social -4.14, p=0.05  RAND vitality 0.45, p=0.98  RAND physical -5.55, p=0.001  RAND mental -1.59, p=0.08  RAND pain -2.26, p=0.19  RAND general health 1.93, p=0.13  RAND health change -3.04, p=0.05  RAND physical role -1.97, p=0.42  RAND PHS -1.97, p=0.17  RAND MHS -2.61, p=0.04  24 M: RAND emotional role -3.38, p=0.37  RAND social -4.46, p=0.13  RAND vitality -0.49, p=0.84  RAND physical -4.39, p=0.06  RAND mental -2.54, p=0.22  RAND pain -4.53, p=0.05  RAND general health 1.19, p=0.44  RAND health change -6.90, p=0.02  RAND physical role -3.32, p=0.33  RAND PHS -2.77, p=0.17  RAND MHS -2.76, p=0.25 | No |
